# Comparative Assessment of Physical and Chemical Cyanobacteria Cell Lysis Methods for Total Microcystin-LR Analysis

**DOI:** 10.3390/toxins13090596

**Published:** 2021-08-26

**Authors:** Katherine E. Greenstein, Arash Zamyadi, Eric C. Wert

**Affiliations:** 1 Southern Nevada Water Authority (SNWA), P.O. Box 99954, Las Vegas, NV 89193-9954, USA; katie.greenstein@gmail.com; 2 Civil, Geological and Mining Engineering Department, Polytechnique Montréal, Montreal, QC H3C 3A7, Canada; Arash.Zamyadi@waterra.com.au; 3 Population Health and Immunity Division, Walter and Eliza Hall Institute of Medical Research (WEHI), 1G, Royal Parade, Parkville, Melbourne, VIC 3052, Australia; 4 Water Research Australia (WaterRA), 990 La Trobe Str., Docklands, Melbourne, VIC 3008, Australia

**Keywords:** cyanotoxin, *Microcystis aeruginosa*, sonication, freeze-thaw, microwave, Abraxis QuikLyse^TM^, copper sulfate

## Abstract

Standardization and validation of alternative cell lysis methods used for quantifying total cyanotoxins is needed to improve laboratory response time goals for total cyanotoxin analysis. In this study, five cell lysis methods (i.e., probe sonication, microwave, freeze-thaw, chemical lysis with Abraxis QuikLyse^TM^, and chemical lysis with copper sulfate) were assessed using laboratory-cultured *Microcystis aeruginosa* (*M. aeruginosa*) cells. Methods were evaluated for destruction of cells (as determined by optical density of the sample) and recovery of total microcystin-LR (MC-LR) using three *M. aeruginosa* cell densities (i.e., 1 × 10^5^ cells/mL (low-density), 1 × 10^6^ cells/mL (medium-density), and 1 × 10^7^ cells/mL (high-density)). Of the physical lysis methods, both freeze-thaw (1 to 5 cycles) and pulsed probe sonication (2 to 10 min) resulted in >80% destruction of cells and consistent (>80%) release and recovery of intracellular MC-LR. Microwave (3 to 5 min) did not demonstrate the same decrease in optical density (<50%), although it provided effective release and recovery of >80% intracellular MC-LR. Abraxis QuikLyse^TM^ was similarly effective for intracellular MC-LR recovery across the different *M. aeruginosa* cell densities. Copper sulfate (up to 500 mg/L Cu^2+^) did not lyse cells nor release intracellular MC-LR within 20 min. None of the methods appeared to cause degradation of MC-LR. Probe sonication, microwave, and Abraxis QuikLyse^TM^ served as rapid lysis methods (within minutes) with varying associated costs, while freeze-thaw provided a viable, low-cost alternative if time permits.

## 1. Introduction

Cyanotoxins, produced by cyanobacteria, present a risk to drinking water as recognized by health advisory levels established in the United States (U.S.) and several other countries [1,2,3]. Cyanobacterial blooms commonly occur and are expected to increase in frequency and magnitude in the future [4,5,6]. Monitoring plans may include total, intracellular, and extracellular cyanotoxin measurements to assess concentrations already dissolved in the water supply (extracellular) and how much additional risk may be experienced following cell death (total/intracellular). During the water-treatment process, cells and corresponding intracellular cyanotoxins are often removed through sedimentation and filtration [7,8]. However, oxidation processes may result in either complete or partial cell lysis [9,10]. Adsorption processes have also been shown to cause shearing of cells, potentially resulting in the release of intracellular cyanotoxins [11]. Due to potential for release of intracellular cyanotoxins in either the source water or the treatment process, drinking-water utilities must consider total cyanotoxin concentrations for appropriate planning and risk management.

Conventional analytical methods (i.e., enzyme-linked immunoassay (ELISA), liquid chromatography-mass spectrometry (LC-MS)) are often used to measure extracellular cyanotoxin concentration. Extracellular cyanotoxins are determined by prefiltering a sample to remove cells and other debris prior to analysis. Total cyanotoxin (i.e., combined intracellular and extracellular concentrations) analysis requires additional sample processing to lyse cells and release intracellular cyanotoxins prior to analysis. A wide variety of cell lysis methods are employed in literature, including lyophilization, bead beating, microwave, autoclaving, boiling, freeze-thaw, sonication, and chemical lysis. While the freeze-thaw process is frequently considered a standard method to lyse cells [12], it can require more time than other methods and may not recover all intracellular cyanotoxins present in samples [13,14,15]. There is a need to standardize and validate alternative cell lysis methods used for quantifying total cyanotoxins in order to give water-quality providers and laboratories more options to meet their response time goals for total cyanotoxin analysis.

In this study, five cell lysis methods (i.e., probe sonication, microwave, freeze-thaw, chemical lysis with Abraxis QuikLyse^TM^, and chemical lysis with copper sulfate) were assessed. Methods were evaluated for destruction of cells (as determined by optical density of the sample at 730 nm) and recovery of total microcystin from *Microcystis aeruginosa* (*M. aeruginosa*) cells (analyzed via liquid chromatography-tandem mass spectrometry (LC-MS/MS)). Additionally, the cost and time required for employing each method were compared.

## 2. Results

### 2.1. Probe Sonication

**Figure 1:**
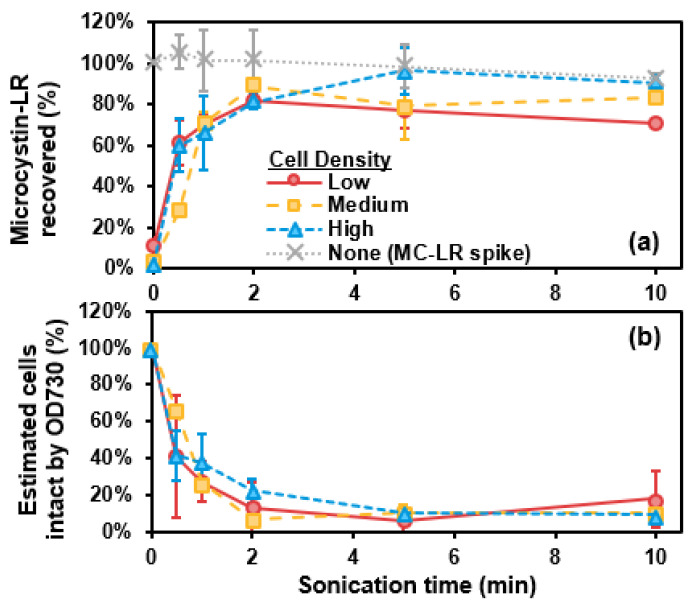
(**a**) Microcystin-LR (MC-LR) recovery (%) and (**b**) estimated cells remaining intact (by optical density at 730 nm, OD730) (%) versus sonication time (minutes). Error bars represent standard deviation from duplicate experiments. Data are shown for low (~10^5^ cells/mL, red circles), medium (~10^6^ cells/mL, yellow squares), and high (~10^7^ cells/mL, blue triangles) density suspensions of laboratory-cultured *M. aeruginosa* in phosphate buffer. The MC-LR-spiked control solution (with no cells) is represented by the grey dotted line.

Recovery of total microcystin-LR (MC-LR) (as percentage of total MC-LR, determined for each cell suspension by analyzing a sample after three freeze-thaw cycles) and estimated cells intact by optical density at 730 nm (OD730) (as a percentage of total cells present before sonication) versus sonication time are shown in Figure 1. By 2 min, approximately all MC-LR (~5, 20, and 40 µg/L for low, medium, and high cell-density suspensions in phosphate buffer, respectively) was extracellular and thus measurable as total MC-LR (Figure 1a). Additionally, OD730 indicated that most cells were destroyed by 2 min (Figure 1b). As a control, the ~15 µg/L MC-LR concentration (as a spike, without cells) did not change throughout 10 min of sonication (Figure 1a). Additionally, 10 min of sonication of cells in Colorado River water (CRW) likewise made approximately all MC-LR extracellular and destroyed most cells (Figure S2). Similarly, approximately all MC-LR was recovered in the control in CRW. Cross-laboratory validation of sonication also demonstrated that most cells were destroyed, and the majority of MC-LR was extracellular by 2 min (Figure S3). While MC-LR recovery slightly fluctuated across 0.5 to 10 min, the concentrations present in the cross-laboratory suspensions were much lower, near the LC-MS/MS method detection limit (MDL) of 0.5 µg/L (Figure S3c). For the cross-laboratory control, the ~2 µg/L MC-LR concentration (as a spike, without cells) did not change throughout 10 min of sonication (Figure S3b).

### 2.2. Microwave

**Figure 2:**
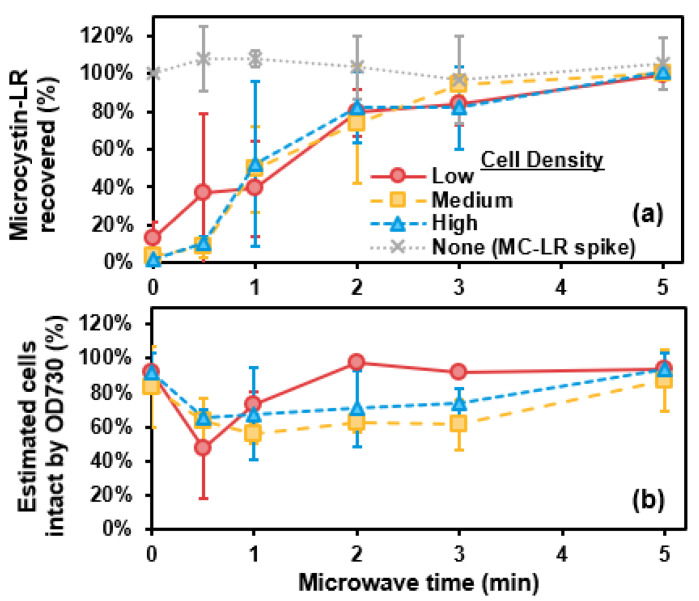
(**a**) Microcystin-LR (MC-LR) recovery (%) and (**b**) estimated cells remaining intact (by optical density at 730 nm, OD730) (%) versus microwave time (minutes). Error bars represent standard deviation from duplicate experiments. Data are shown for low-, medium-, and high-density suspensions of laboratory-cultured *M. aeruginosa* in phosphate buffer. The MC-LR-spiked control solution (with no cells) is represented by the grey dotted line.

Recovery of MC-LR (as percentage of total MC-LR) and estimated cells intact by OD730 (as percentage of total cells present before microwave) versus microwave time are shown in Figure 2. Volume losses from microwave varied from vial to vial, ranging from 0 to 3 mL (0 to 30% of sample volume) lost, requiring each MC-LR concentration to be adjusted accordingly. While most samples had volume loss, even at shorter (0.5 min) microwave times, sample loss predominantly increased with longer microwave times. Approximately all MC-LR was extracellular and thus measurable as total MC-LR with 2 min of microwave (Figure 2a). However, estimated cells by OD730 remained approximately the same throughout 5 min of microwave (Figure 2b). As a control, MC-LR concentration (as a spike, without cells) did not change throughout 5 min of microwave (Figure 2a). Recovery of MC-LR from cells in CRW via 5 min of microwave was relatively low, at ~60 (±14)% (Figure S4), when compared with recoveries in phosphate buffer. This corresponded with significant sample volume loss of 2.8 mL (28%). The MC-LR control was recovered at 85 (±9)%, with sample volume loss of 3 mL (30%).

### 2.3. Freeze-Thaw

**Figure 3:**
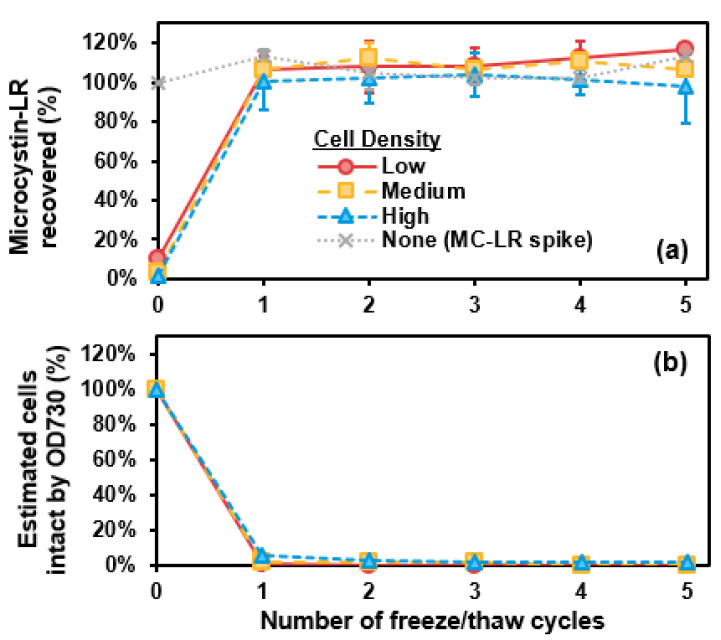
(**a**) Microcystin-LR (MC-LR) recovery (%) and (**b**) estimated cells remaining intact (by optical density at 730 nm, OD730) (%) versus number of freeze-thaw cycles. Error bars represent standard deviation from duplicate experiments. Data are shown for low-, medium-, and high-density suspensions of laboratory-cultured *M. aeruginosa* in phosphate buffer. The MC-LR-spiked control solution (with no cells) is represented by the grey dotted line.

Recovery of MC-LR (as percentage of total MC-LR) and estimated cells intact by OD730 (as percentage of total cells present before freeze/thaw) versus freeze/thaw cycles are shown in Figure 3. All MC-LR was extracellular and thus measurable as total MC-LR after one freeze/thaw cycle (Figure 3a). All cells were destroyed by one freeze/thaw cycle (Figure 3b). As a control, MC-LR concentration (as a spike, without cells) did not change throughout 5 cycles of freeze/thaw (Figure 3a). Additionally, for cells in CRW, 5 cycles of freeze-thaw released all MC-LR as extracellular and destroyed most cells (Figure S5). For the control in CRW, MC-LR was also fully recovered.

### 2.4. Abraxis QuikLyse^TM^

**Figure 4:**
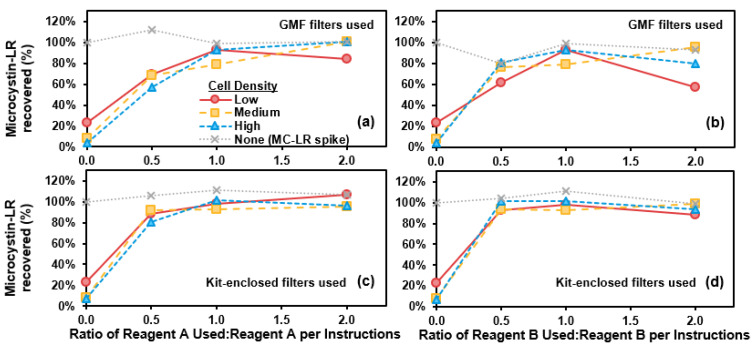
Microcystin-LR (MC-LR) recovery (%) versus (**a**,**c**) ratio of Abraxis QuikLyse^TM^ Reagent A used relative to manufacturer instructions or (**b**,**d**) ratio of Abraxis QuikLyse^TM^ Reagent B used relative to manufacturer instructions. For (**a**,**b**), glass microfiber filters were used to filter samples, while for (**c**,**d**), Abraxis kit-enclosed filters were used. Data are shown for low-, medium-, and high-density suspensions of laboratory-cultured *M. aeruginosa* in phosphate buffer. The MC-LR-spiked control solution (with no cells) is represented by the grey dotted line.

Recovery of MC-LR (as percentage of total MC-LR) versus Reagent A or Reagent B volumes (0.5×, 1×, or 2× manufacturer instructions) are shown in Figure 4. For either GMF syringe filters or kit-enclosed filters, all MC-LR was extracellular and thus measurable as total MC-LR at the reagent: sample ratios prescribed in the Abraxis QuikLyse^TM^ instructions (1× the reagent volumes in the instructions). Using kit-enclosed filters, lower reagent:sample ratios (0.5× Reagent A or Reagent B, with 1× of the other respective reagent) also appeared to yield recovery of approximately all MC-LR (Figure 4c,d). OD730 increased with use of reagents and was not representative of cell counts, as a white precipitate formed upon addition of Reagent B to the solutions (e.g., Figure S6). The kit-enclosed filters allowed some precipitate to pass (while the GMF filters did not), and so kit-enclosed-filtered samples were allowed to settle in a refrigerator for one week prior to analysis of MC-LR via LC-MS/MS. As a control, MC-LR concentration (as a spike, without cells) did not change with the addition of different volumes of reagents (Figure 4a,b). In addition, most MC-LR was released to extracellular with the application of 1× reagent volumes on cells suspended in CRW (Figure S7). For the control in CRW, all MC-LR was recovered.

### 2.5. Copper Sulfate

**Figure 5:**
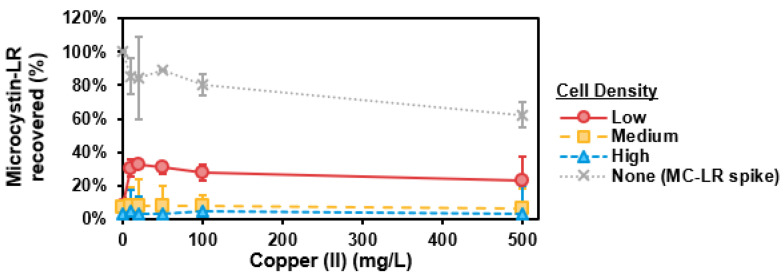
Microcystin-LR (MC-LR) recovery (%) versus copper sulfate (as Cu^2+^) concentration (mg/L). Error bars represent standard deviation from duplicate experiments. Data are shown for low-, medium-, and high-density suspensions of laboratory-cultured *M. aeruginosa* in phosphate buffer. The MC-LR-spiked control solution (with no cells) is represented by the grey dotted line.

Recovery of MC-LR (as percentage of total MC-LR) versus copper (II) (Cu^2+^) concentration (mg/L) (with 20 min exposure time) is shown in Figure 5. MC-LR was generally not released by cells during the short-term exposure. As a control, MC-LR concentration (as a spike, without cells) did not change through the addition 100 mg/L Cu^2+^ and was measured as minimally lower at 500 mg/L Cu^2+^. Cu^2+^ did not cause release of MC-LR for cells suspended in CRW within 20 min.

## 3. Discussion

### 3.1. Microcystin-LR Recovery

For unicellular, laboratory-cultured *M. aeruginosa* in phosphate buffer, multiple methods were effective for recovering total MC-LR. Probe sonication for 2 min, microwave for 3 min, freeze-thaw for 1 cycle, and chemical lysis with use of 1 × Abraxis QuikLyse^TM^ reagents all similarly made most MC-LR extracellular. As each method has been used previously in cyanobacteria/cyanotoxin studies, each method would be expected to recover MC-LR successfully with optimal conditions, which herein included the presence of discrete spherical unicells (rather than colonies or other cell morphologies). Probe sonication, freeze-thaw, and 1 × Abraxis QuikLyse^TM^ remained effective for MC-LR recovery when cells were suspended in a natural water matrix, CRW.

While probe sonication adequately recovered total MC-LR, it also notably left MC-LR intact without decaying the congener through 10 min of sonication (Figure 1a, Figure S3b). While some studies have referred to degradation of MCs from sonication, ultrasonic baths [16,17] or probe sonicators [18] were used without cooling the samples during processing. An ultrasonic bath was previously shown to degrade MC-LR in solution without cells [17]. However, the optimized conditions used in this study—pulsed sonication (5 s on and 1 s off) and samples kept in an ice bath during sonication—specifically ensured the sonicator tip and sample remained at or below room temperature. This likely prevented the MC-LR degradation observed in other studies and maintains the importance of quality assurance/quality control during cell lysis of samples as described in drinking water methods for analyzing MCs [12,19]. Another study also demonstrated greater MC recovery from dense bloom samples with a probe sonicator applied for 5 min when compared with three freeze-thaw cycles even when followed by 30 min in an ultrasonic bath [13]. This further highlights the difference between probe sonication versus ultrasonic bath, with probe sonication (with a tip appropriately sized for sample volume) most effectively recovering intracellular cyanotoxins.

Microwave for 3 to 5 min achieved adequate MC-LR recovery in most samples. This was relatively consistent with other studies that demonstrated 9 to 20 min of microwave enabled recovery of total MCs [15,18]. However, recovery of MC-LR by microwave for cells suspended in CRW was lower than that applied to cells in phosphate buffer, and recovery of MC-LR in the control CRW showed greater variation when compared with the other lysis methods. Further, recovery of MC-LR via microwave in phosphate buffer also exhibited greater variation when compared with the other methods (as shown by the standard deviation error bars in Figure 2a). This was likely due to error introduced by volume loss and corresponding adjustment of MC-LR concentrations, which can be exacerbated when sample volumes (and measurements of sample volume loss) are relatively small. Such variation from microwave should be addressed by using sealed containers (able to withstand pressure) to hold samples during microwave, though this practice can limit sample volume relative to other methods [18]. Additionally, microwave without allowing samples to boil can minimize sample loss (and potential associated error) [15]. Microwave for 10 to 20 min has been shown to be more effective for MC recovery than three freeze-thaw cycles in natural bloom samples, and microwave lysis does not degrade multiple MC congeners [15].

Although even one cycle of freeze-thaw was effective and consistent for recovering MC-LR from unicellular *M. aeruginosa*, other studies indicate freeze-thaw is not always effective in recovering all MCs in different types of samples. Freeze-thaw for three cycles or use of Abraxis QuikLyse^TM^ have sometimes recovered comparable amounts of MCs in natural bloom samples [20], but in other cases, freeze-thaw has yielded irreproducible or low MC recoveries [13,14]. This variation appears linked to one or more differences in the samples, including cell age, density of cells in sample, sample volume, and/or species present [13,14,20].

For chemical lysis, Abraxis QuikLyse^TM^ was comparably effective for recovering MC-LR when compared with other methods, including freeze-thaw, as has been shown in another study [20]. In contrast, copper sulfate as a chemical lysing agent did not recover MC-LR within 20 min, a similar time period employed by chemical lysis via Abraxis QuikLyse^TM^. This is consistent with literature, as copper sulfate typically lysed cells on the scale of hours to days [21,22,23]. Based on differences observed between cell lysis methods from the collective body of literature, further study would be beneficial for evaluating if a variety of sample factors could impact MC recovery via Abraxis QuikLyse^TM^.

### 3.2. Cell Destruction

As another indicator of cell lysis efficacy, cell destruction was evaluated across the different lysis methods. Cell destruction was estimated using OD730, which indicated laboratory-cultured *M. aeruginosa* unicells were fragmented via 2 min of sonication or one freeze-thaw cycle. However, OD730 remained relatively unchanged throughout microwave up to 5 min and could not be estimated for Abraxis QuikLyse^TM^, which formed a precipitate in samples. While cell destruction could not be estimated for copper sulfate via OD730 due to particulate copper present, the lack of release of intracellular MC-LR suggests cell damage may not have occurred within 20 min of exposure.

Probe sonication typically achieves destruction of 70% or more cells for both laboratory-cultured and naturally occurring cyanobacteria [24]. Additionally, probe sonication degrades chlorophyll-*a*, which impacts OD730 measurements. Ultrasonic baths can similarly destroy up to 70% of cells [16], but that does not always occur. For example, sonication (in a water bath) for 60 min only destroyed the most external cells of colonies, and *Nodularia* cells were destroyed more readily than those of freshwater *Microcystis* and *Oscillatoria*, highlighting how cell morphology can impact lysis [13]. However, in the same study, probe sonication was shown to successfully disrupt cells, and additional literature supports that probe sonication fragments cells and disrupts colonies [14,25]. Bead-beating, a similarly physical method used to disrupt cells, has been reported to destroy a majority of cells [16,26,27], like probe sonication. However, probe sonication does not require adding consumables, like glass or metal beads, prior to sample agitation.

While OD730 did not confirm cell destruction by microwave, another study demonstrated that after microwave for 6 to 9 min, future cell regrowth was prevented, and samples were effectively sterilized, indicating cells were damaged [18]. Within a broader category of “thermal” cell lysis, autoclaving and boiling cells have been shown to moderately disrupt colonies and cells [24]. Based on the successful MC recovery previously observed via microwave when boiling of samples was avoided, microwave is able to lyse cells despite not causing complete destruction of cell morphology [15].

Freeze-thaw, while extremely effective at destroying unicellular *M. aeruginosa* with one cycle as estimated by OD730, only destroyed 19% of cells under similar experimental conditions in another study as determined by cell counting [16]. Additionally, while freeze-thaw cycles can destroy colonies (more so than autoclaving or boiling in certain conditions), the process can result in less chlorophyll-*a* degradation than thermal lysis methods [24]. Multiple freeze-thaw cycles, such as the three cycles prescribed in EPA Method 546 [12], appear necessary for natural bloom samples, as one freeze-thaw cycle may only disrupt a small number of colonies [13].

Although OD730 did not indicate cell destruction due to the precipitate formed during chemical lysis with Abraxis QuikLyse^TM^, other studies have evaluated impacts to cells from use of the reagents. Abraxis QuikLyse^TM^, when compared with freeze-thaw, boiling, autoclaving, and probe sonication, was shown to be the least effective at destroying cells (as visible by light microscopy with staining), even with use of reagents at three times the volume of manufacturer instructions [24]. The chemical reagents were capable of degrading some colonies and filaments, yielding higher numbers of single cells after exposure, and some chlorophyll-*a* was also degraded. Further exploration of cell lysis (and corresponding MC recovery) using Abraxis QuikLyse^TM^ reagents could elucidate the efficacy of the chemicals in lysing cells in naturally occurring bloom samples.

OD730 did not correlate with cell density upon the introduction of particulate copper when copper sulfate was applied; however, with no intracellular MC-LR recovered, it is not expected that cells were lysed within the 20 min of exposure. Cell lysis from copper sulfate likely would have occurred in hours or days based on literature [21,22,23].

### 3.3. Time, Cost, and Practicality

Freeze-thaw is a relatively inexpensive method for cell lysis and can be conducted solely using a freezer. However, freeze-thaw cycles can take hours or longer, which can prevent completing rapid analysis for cyanotoxins. Limiting sample volume and using a warm water bath for thawing samples can help expedite the process. Three freeze-thaw cycles are included as the process for cell lysis in EPA Method 546 [12].

Probe sonication involves a relatively high initial capital cost when compared with other cell lysis methods (e.g., $5775 in 2021 for the QSonica 500, ¼ inch microtip, converter holder, and sound enclosure used herein). However, after acquisition of a probe sonicator, operating costs are minimal, and cells can be lysed within minutes. Additionally, probe sonicators may be outfitted with horns that have multiple tips, which would enable simultaneous sonication of multiple samples.

Microwave, like freeze-thaw, is an inexpensive method for cell lysis. While quicker than freeze-thaw, measuring remaining sample volume and ensuring proper adjustment of concentration can take additional time (e.g., relative to probe sonication). Use of sealed containers during microwave [18], which would prevent volume loss in samples, can decrease result variability and time required. However, this practice limits sample volume and requires additional special considerations for sample container.

Abraxis QuikLyse^TM^, while at 40 min, is slower than probe sonication but faster than freeze-thaw, introduces a considerable operating cost relative to the other methods examined herein. At $87.20 in 2021 per kit (for 45 samples that are 1 mL in volume each), it also requires consideration of how rapidly it will be used (as it has an expiration date) and limits the sample volume that can be processed. As copper sulfate did not lyse cells within a similar timeframe as Abraxis QuikLyse^TM^, it was not given further consideration for chemical lysis.

## 4. Conclusions

Of the physical lysis methods, both freeze-thaw (one to five cycles) and pulsed probe sonication (2 to 10 min) most clearly resulted in destruction of laboratory-cultured cells and consistent release of intracellular MC-LR. While microwave and Abraxis QuikLyse^TM^ did not demonstrate the same decrease in OD730 correlated with cell destruction in this study, these methods also resulted in effective release of intracellular MC-LR. Concentrations of copper sulfate (up to 500 mg/L Cu^2+^) did not lyse cells within 20 min. Probe sonication holds promise as a rapid (within minutes) method, with no decay of MC-LR observed, though additional study of impacts to other MC congeners is merited. Freeze-thaw represents a viable, low-cost alternative if time permits.

While microscopy suggests sonication and freeze-thaw lysis methods can similarly degrade filamentous cyanobacteria and colonial *Microcystis* [24], additional method development and validation quantifying toxin release is needed for different cell morphologies, densities, and natural cells. Natural cells, which frequently consist of mixtures of cyanobacterial species and morphologies that employ a protective sheath, have been shown to resist oxidative treatment better than laboratory-cultured cells [28]. Similarly, natural cells may necessitate more rigorous lysis conditions to achieve complete intracellular cyanotoxin release. Relatively higher resilience of cells, depending on age, morphology, density, and/or other sample characteristics, appears to have impacted freeze-thaw lysis studies to date. One or more freeze-thaw cycles have not consistently resulted in complete cell destruction [13,16] or as much chlorophyll-*a* degradation when compared with other methods (e.g., probe sonication) [24].

Further, most work has centered on microcystin-producing cyanobacteria and MC-LR. Evaluation of lysis methods for cyanobacteria producing emerging cyanotoxins, including other MC congeners, cylindrospermopsin, anatoxin, and saxitoxin, is needed in light of evolving health concerns and water-monitoring capabilities.

## 5. Materials and Methods

### 5.1. Cyanobacteria Culturing and Standard Preparation

*Microcystis aeruginosa* (unicellular, LB 2385, UTEX Culture Collection of Algae at the University of Texas at Austin, hereafter “*M. aeruginosa*”) was confirmed to produce only microcystin-LR (MC-LR) and cultured as described in previous work [29]. After 30 days of culture growth, cells were centrifuged and rinsed with 1 mM phosphate buffer adjusted to pH 7.5 (hereafter, phosphate buffer) three times and ultimately suspended in phosphate buffer at concentrations of 1 × 10^5^ cells/mL (low-density), 1 × 10^6^ cells/mL (medium-density), and 1 × 10^7^ cells/mL (high-density) for cell lysis experiments. The concentration of *M. aeruginosa* cells in the stock suspension was confirmed by counting cells using digital flow cytometry as described in previous work [30]. The cell densities corresponded with approximately 5, 20, and 40 µg/L total MC-LR, respectively (verified for each batch of cell suspension via analysis of a sample subjected to three freeze-thaw cycles).

Additionally, a known concentration of MC-LR (Enzo Life Sciences, ≥95%), ~15 µg/L, was spiked into phosphate buffer as a control solution to ensure cyanotoxin recovery.

### 5.2. Cell Lysis

For cell lysis experiments, 10 mL volumes of *M. aeruginosa* suspensions were each placed in 40 mL amber glass vials with Teflon-lined caps. Samples were then subjected to sonication, microwave, freeze-thaw, Abraxis QuikLyse^TM^ reagents, or copper sulfate. All experiments were performed in duplicate. Experiments were conducted at Southern Nevada Water Authority unless specifically indicated otherwise.

#### 5.2.1. Probe Sonication

A sonicator (QSonica Q500, Newton, CT, USA) fitted with a ¼ inch probe microtip was used for cell lysis via sonication. The microtip applied an amplitude of 200 µm to samples, with a power of approximately 30 W at a frequency of 20 kHz. The microtip was designed to process volumes of 5 mL up to 50 mL. For experiments, 40 mL vials containing 10 mL of either low-, medium-, or high-density cell suspension (or phosphate buffer spiked with MC-LR as control) were individually placed in an ice bath to prevent overheating of the solution and/or damage to the sonicator probe tip. Temperature measurements before and after sonication confirmed samples remained at 25 °C or less. The probe tip was lowered halfway into sample solution to ensure adequate submersion while preventing glass breakage during sonication. Sonication was pulsed (5 s on, 1 s off) and applied to samples for 30 s, 1 min, 2 min, 5 min, or 10 min. The probe tip was cleaned with ethanol and DI water between sonication of different vials.

#### 5.2.2. Microwave

A microwave (Kenmore Stock No. 86951, Chicago, IL, USA) was used for cell lysis via microwave. For experiments, 10 mL of low-, medium-, or high-density cell suspension (or phosphate buffer spiked with MC-LR as control) were individually placed in 100 mL Erlenmeyer flasks, set within a larger glass beaker, and covered with a large Kimwipe^®^. Samples were microwaved at 10% power (to prevent loss of all sample volume) for 30 s, 1 min, 2 min, 3 min, or 5 min. Sample volumes were quantified after microwaving with a graduated cylinder to account for loss and adjust MC-LR concentrations accordingly. Samples were immediately placed on ice to rapidly cool to room temperature.

#### 5.2.3. Freeze-Thaw

A freezer maintaining a temperature of −20 °C was used for cell lysis via freeze-thaw, while thawing was conducted at room temperature, 25 °C. For experiments, 40 mL vials containing 10 mL of either low-, medium-, or high-density cell suspension (or phosphate buffer spiked with MC-LR as control) were individually placed on their sides in the freezer (to prevent glass breakage) overnight (~12 h) and subsequently placed on a countertop until thawed at room temperature (2 to 4 h). For some samples, this process was repeated to test up to five freeze-thaw cycles.

#### 5.2.4. Abraxis QuikLyse^TM^

Abraxis QuikLyse^TM^ kits (Eurofins Abraxis, Warminster, PA, USA) were tested as a chemical lysis process. Chemical reagents from the kit (“Reagent A” and “Reagent B”) were added in the sequence and at the times prescribed by manufacturer instructions to a 40-mL vial containing 10 mL of the cell suspension (or phosphate buffer spiked with MC-LR as control). For reagent volumes, either the manufacturer-instructed volume ratios (1 or less or more of either Reagent A or Reagent B (0.5× or 2×) were used. While Reagent A or Reagent B was varied in volume ratio used, the other reagent was applied at the manufacturer-instructed volume ratio (1×).

#### 5.2.5. Copper Sulfate

Use of copper sulfate was tested as a chemical lysis process. Copper sulfate was prepared as a 1000 mg/L ionic copper (Cu^2+^) solution, and known concentrations of copper sulfate (10, 20, 50, 100, or 500 mg/L as Cu^2+^) were spiked into 40 mL vials each containing 10 mL of cell suspension (or phosphate buffer spiked with MC-LR as control). Vials were placed on shake tables to ensure adequate mixing of the solution for 20 min.

### 5.3. Matrix Evaluation

A high-density (1 × 10^7^ cells/mL) suspension was also prepared in CRW (DOC of 2.5 mg/L, pH 8, alkalinity of 138 mg/L) to further test 10 min of sonication, 5 min of microwave, 5 freeze/thaw cycles, instruction-based (1×) reagent volumes of Abraxis QuikLyse^TM^, or 500 mg/L Cu^2+^. Additionally, a known concentration of MC-LR (~15 µg/L) was spiked into CRW as a control solution to ensure cyanotoxin recovery.

### 5.4. Post-Lysis Sample Processing

Optical density at 730 nm (OD730) was measured before and after cell lysis using a Hach spectrophotometer and plastic cuvettes with 1-cm path length. The concentration of intact cells was correlated to OD730, as shown in Figure S1 in Supplemental Information (SI).

Immediately following cell lysis and measurement of OD730, samples were filtered for MC-LR analysis using 10-mL glass syringes and glass microfiber (GMF) syringe filters (Whatman^®^, diameter of 25 mm, pore size of 0.45 µm). For each sample, at least 1 mL of sample was passed through the filter to waste prior to collection of approximately 2 mL of filtered sample in a 10-mL amber glass vial for subsequent analysis. As an exception, for some of the Abraxis QuikLyse^TM^ samples, kit-enclosed filters, which utilized disposable plastic pipettes and plastic pipette tips containing a filter material, were used to filter samples as per manufacturer instructions. Following the chemical lysis, samples were immediately analyzed for optical density and either filtered with kit-enclosed filter tips and disposable pipettes or with glass syringes and GMF syringe filters for MC-LR analysis. Subsequently, samples were taken for optical density analysis and filtered for MC-LR analysis using glass syringes and glass microfiber GMF syringe filters. Filtered samples were refrigerated and analyzed for MC-LR within two weeks via LC-MS/MS as described in previous work [31].

### 5.5. Cross-Laboratory Validation

A probe sonicator was used with equivalent settings at a separate laboratory (Polytechnique Montréal) to validate sonication results using laboratory-cultured MC-LR-producing *M. aeruginosa* (unicellular, CPCC 300, Canadian Phycological Culture Centre) suspended in 1 mM phosphate buffer adjusted to pH 7.5. Cell culturing and the probe sonication apparatus were as described in previous work [32]. Suspensions were either low density (~3 × 10^6^ cells/mL) or medium density (~7 × 10^6^ cells/mL), corresponding with ~0.7 or ~1 µg/L MC-LR, respectively (verified for each batch of cell suspension via analysis of a sample subjected to three freeze-thaw cycles). Phosphate buffer spiked with MC-LR (Abraxis, ≥95%) at a concentration of ~2 µg/L (with no cells present) was used as a control. Samples were processed and analyzed for estimated cell count and MC-LR concentration as previously described.

## Data Availability

The data presented in this study are available in the article and supplementary information.

## Supplementary Materials

The following are available online in Supplemental Information at https://www.mdpi.com/article/10.3390/toxins13090596/s1: Figure S1: correlation of cell count versus optical density at 730 nm; Figure S2: estimated cells intact and MC-LR recovered versus sonication time for cells in CRW; Figure S3: estimated cells intact and MC-LR recovered versus sonication time for cells tested during cross-laboratory validation; Figure S4: MC-LR recovered versus microwave time for cells in CRW; Figure S5: estimated cells intact and MC-LR recovered versus freeze-thaw cycles for cells in CRW; Figure S6: images of precipitate formed when using Abraxis QuikLyse^TM^; Figure S7: MC-LR recovered versus use of Abraxis QuikLyse^TM^ reagents.
