# Supplementary material for: Comparative Assessment of Physical and Chemical Cyanobacteria Cell Lysis Methods for Total Microcystin-LR Analysis
Source: Toxins (Basel). 2021 Aug 26;13(9):596. doi: 10.3390/toxins13090596 (PMC8473049; doi:10.3390/toxins13090596)
Supplement: Supplementary file 1 [file toxins-13-00596-s001.zip › toxins-1335747-SI.pdf]

Comparative Assessment of Physical and Chemical Cyanobacteria Cell Lysis Methods for Total Microcystin-LR Analysis

Katherine E. Greenstein, Arash Zamyadi and Eric C. Wert

MATERIALS AND METHODS

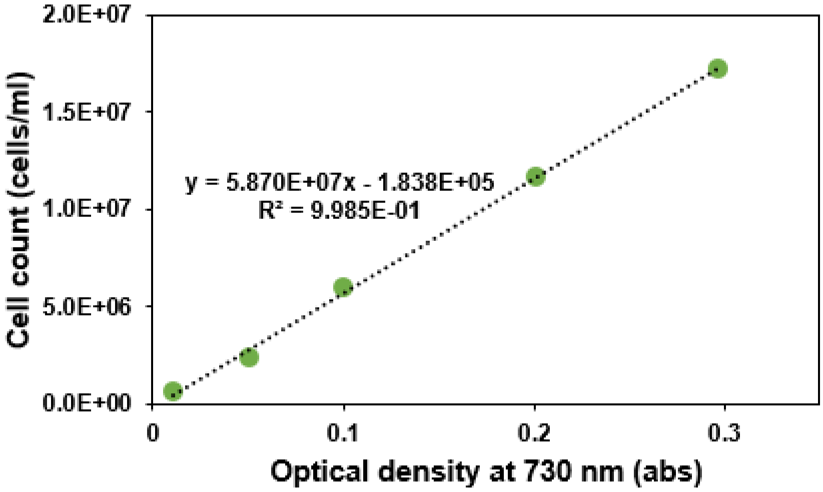

Figure S1. Correlation of cell count (cells/mL) versus optical density at 730 nm (OD730). Laboratory-cultured *M. aeruginosa* cells were counted using digital flow cytometry.

RESULTS AND DISCUSSION

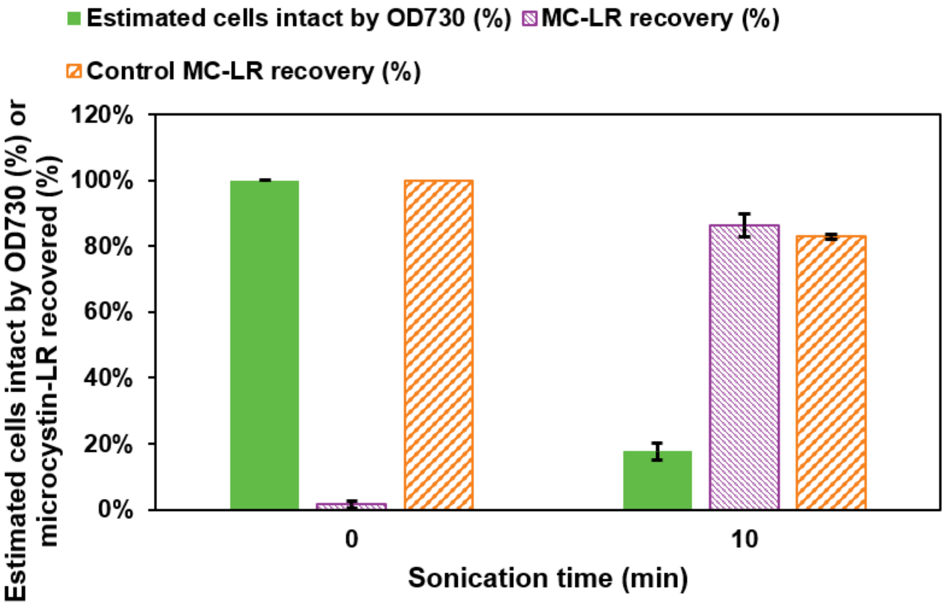

Figure S2. Estimated cells intact by OD730 (%, solid green) or MC-LR recovered (%, striped purple) versus sonication time (minutes). Error bars represent standard deviation from duplicate experiments. Sonication was tested on laboratory-cultured *M. aeruginosa* suspended in Colorado River water (CRW) at high (~10^7 cells/mL) cell density. Recovery of MC-LR from controls in CRW (no cells present) is also shown (%, striped orange).

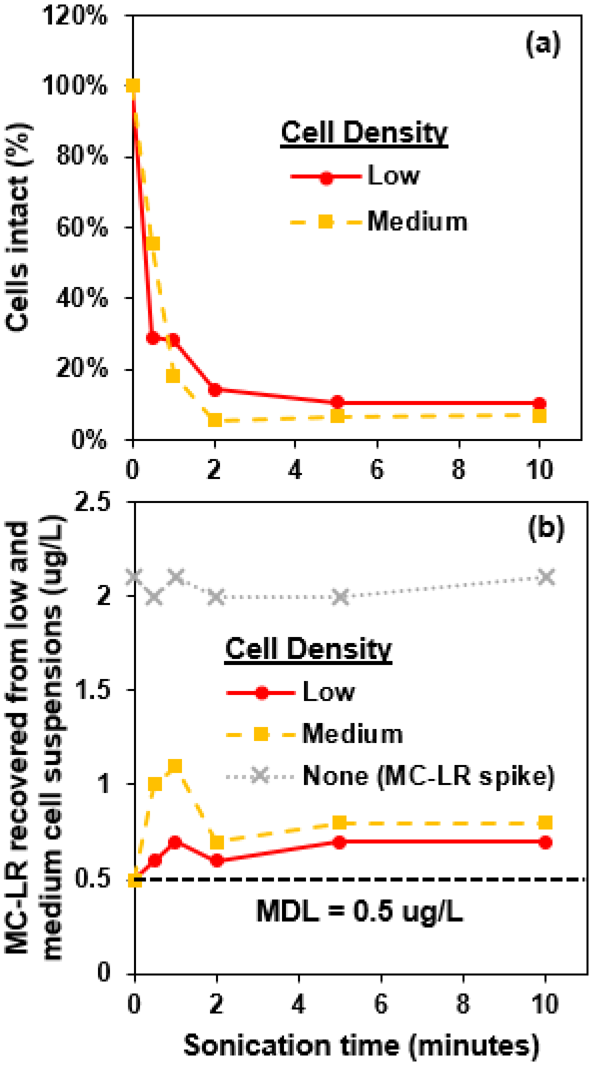

Figure S3. (**a**) Estimated cells remaining intact (%) and (**b**) microcystin-LR (MC-LR) recovery (ug/L) versus sonication time (minutes) for laboratory-cultured *M. aeruginosa* in a Canadian laboratory (Polytechnique Montréal) for cross-laboratory validation. Data are shown for low (~10^5 cells/mL, red circles) and medium (~10^6 cells/mL, yellow squares) density suspensions of laboratory-cultured *M. aeruginosa* in phosphate buffer. The MC-LR-spiked control solution (with no cells) is represented by the grey dotted line.

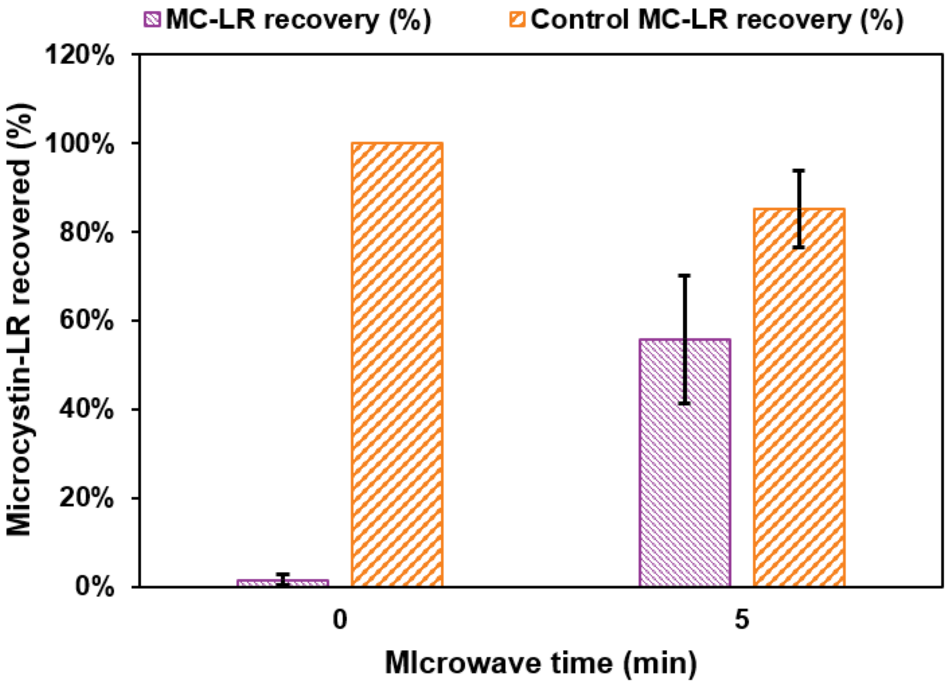

Figure S4. MC-LR recovered (%) versus microwave time (minutes). Error bars represent standard deviation from duplicate experiments. Sonication was tested on laboratory-cultured *M. aeruginosa* suspended in Colorado River water (CRW) at high (~10^7 cells/mL) cell density. Recovery of MC-LR from controls in CRW (no cells present) is also shown (%, striped orange).

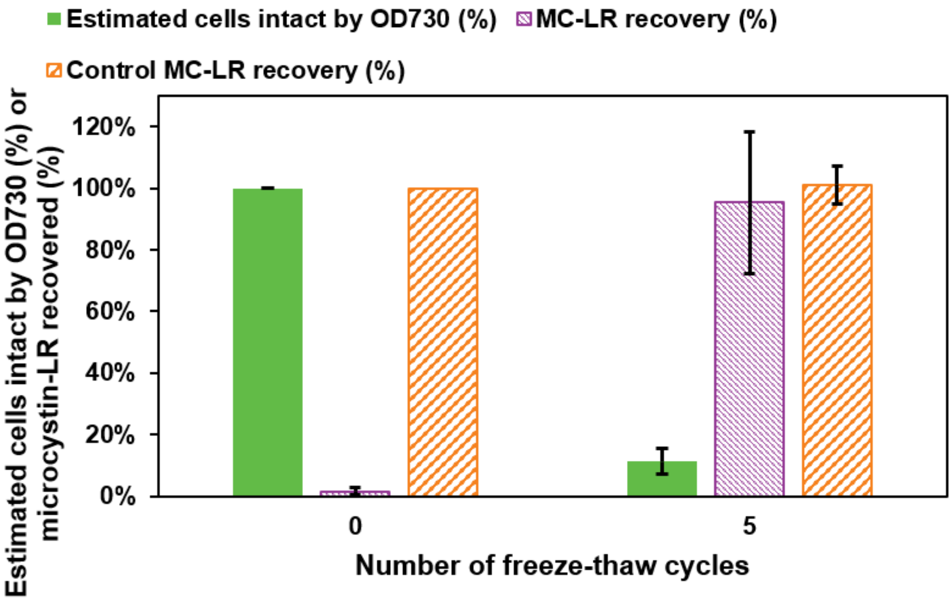

Figure S5. Estimated cells intact by OD730 (%, solid green) or MC-LR recovered (%, striped purple) versus sonication time (minutes). Error bars represent standard deviation from duplicate experiments. Sonication was tested on laboratory-cultured *M. aeruginosa* suspended in Colorado River water (CRW) at high (~10^7 cells/mL) cell density. Recovery of MC-LR from controls in CRW (no cells present) is also shown (%, striped orange).

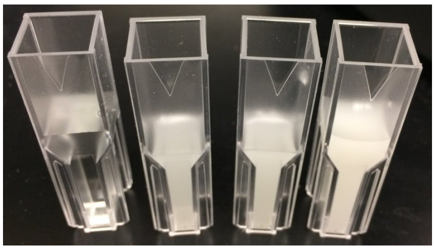

Figure S6. Image of cuvettes containing solution with no Abraxis QuikLyse™ reagents (left) with increasing amounts of reagents as cuvettes progress to the right. A white precipitate formed with the addition of reagents, which necessitated allowing samples to settle, even after filtering with Abraxis kit-enclosed filters, prior to MC-LR analysis via liquid chromatography-tandem mass spectrometry (LC-MS/MS).

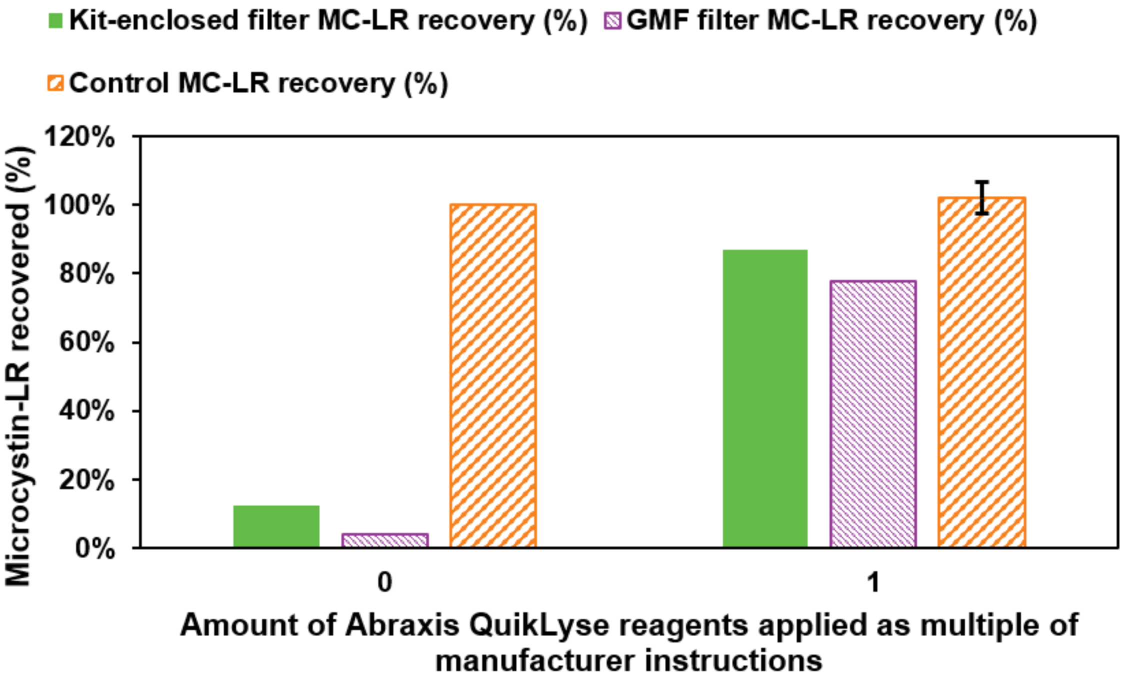

Figure S7. MC-LR recovered (%) versus use of Abraxis QuikLyse™ reagents. Both kit-enclosed filters (solid green) and glass microfiber (GMF) filters (striped purple) were separately examined. The chemical lysis was tested on laboratory-cultured *M. aeruginosa* suspended in Colorado River water (CRW) at high (~10^7 cells/mL) cell density. Recovery of MC-LR from controls in CRW (no cells present) is also shown (%, striped orange).
